# Microcanonical Analysis of Semiflexible Homopolymers with Variable-Width Bending Potential

**DOI:** 10.3390/polym17070906

**Published:** 2025-03-27

**Authors:** Matthew J. Williams, Michael C. Gray

**Affiliations:** School of Engineering, Murray State University, Murray, KY 42071, USA

**Keywords:** semiflexible polymers, microcanonical analysis, replica-exchange Monte Carlo, structural phases

## Abstract

Understanding the structural dynamics of semiflexible polymers in an implicit solvent under varying conditions provides valuable insights into their behavior in diverse environments. In this work, we systematically investigate the effect of the angular width of the bending potential on structural state behavior and conformational variability using microcanonical analysis. A range of angular widths is explored, with the widest value corresponding directly to the classic semiflexible polymer model, which exhibits a diverse set of structural states, including Two-Strand, Three-Strand, Four-Strand, Ring, Random Coil, and Globule configurations. As the angular width narrows, structural variability within states decreases, overlap between structural states is reduced, and conformations become more stable, leading to an expansion of the parameter space dominated by individual structures. By examining microcanonical entropy and its derivatives, we identify transitions analogous to first-, second-, and third-order thermodynamic transitions, providing a deeper understanding of the configurational landscape of semiflexible polymers.

## 1. Introduction

Biopolymers, such as proteins, nucleic acids, and polysaccharides, serve crucial functions within physiological environments, with their roles inherently linked to their structural configurations [1,2,3,4]. Understanding the structural dynamics of these polymer systems under various conditions is crucial in numerous fields, including drug delivery, tissue engineering, and nanomedicine [5,6]. Computer simulations have become essential tools for probing the behaviors of macromolecules. However, while all-atom simulations provide detailed molecular descriptions, they often face computational limitations for large systems and yield case-specific insights, limiting their generality. To address these challenges, coarse-grained effective potential models simplify interactions while preserving essential physical behavior, enabling broader applicability to mesoscopic polymer systems [7,8].

Semiflexible polymer models, like the worm-like chain model [9] and coarse-grained bead-spring models [10], are popular due to their simplicity and ability to approximate biological systems while capturing essential features like bending stiffness. These models enable the representation of a broad range of structural states by adjusting model parameters, energy scales, and incorporating additional effective potentials [11]. Analytical models such as Flory mean-field theory [12] and self-avoiding walk models [13] provide insights into polymer phase transitions in the thermodynamic limit, where the chain length approaches infinity. However, the present study focuses on finite mesoscopic polymer systems, where structural transitions are analyzed within systems of limited size. Unlike models designed for extrapolation to bulk thermodynamic behavior, our approach examines how bending flexibility and angular constraints influence polymer configurations at experimentally relevant mesoscopic scales. Applications include simulations of helical structures [14,15], adsorption on surfaces [16,17], multiple-chain aggregation [18,19], dense packing [20], liquid crystalline ordering in dense polymer solutions [21], crystallization in semiflexible polymer melts [22], and behavior within confined environments [23].

In this study, we introduce a modified semiflexible polymer model that incorporates an angular width parameter, *w*, into a cosine-based bending potential. This parameter influences the structural stability and diversity of the polymers by scaling the range of angular offsets where bending energy is felt. For w=1.0, the model reproduces standard bead-spring semiflexible polymer behavior, while smaller values (e.g., w=0.5) narrow the potential well, leading to enhanced structural stability and expanded dominance of certain structural states. The angular width parameter *w* is introduced specifically for use in coarse-grained polymer models and is not intended for chemically realistic, atomistic-level modeling. While a similar width factor could be introduced for a harmonic bending potential, a change in width would be equivalent to a change in well depth, whereas in the cosine potential, there are two degrees of freedom that can be tuned independently. This versatility offers new opportunities for exploring structural transitions under diverse conditions.

The modified model is simulated using a two-dimensional replica-exchange Monte Carlo approach, which efficiently samples structural configurations for systems exhibiting complex structural transitions [24,25,26]. The histogram reweighting of canonical ensembles allows us to calculate microcanonical entropy, providing thermodynamic markers of structural transitions, including first-, second-, and third-order transitions [27,28].

Our findings highlight the dual role of the angular width parameter in promoting structural stability and diversity, making the variable-width semiflexible polymer model a interesting tool for studying the structural transitions and dynamics of semiflexible polymers.

## 2. Materials and Methods

### 2.1. Model Description

This study employs a coarse-grained semiflexible homopolymer model in which the bending potential includes an angular width parameter which scales the range of angular offsets over which the cosine bending restraint is felt. The energy, *E*, of a polymer chain with *N* monomers includes three potentials: a bonded interaction between adjacent monomers along the polymer chain, a non-bonded interaction between monomers in physical proximity but not directly bonded by the FENE potential, and a bending potential associated with bond angles. In this study, we present data for polymer chains of the lengths N=30 and N=40.

Bonded monomers interact according to the finitely extensible nonlinear elastic (FENE) potential [29]. The FENE potential, presented in Equation (1), depends solely on the distance between the two bonded monomers, *r*. A minimum bond energy is achieved when $r=r_{0}\equiv 1$ and the maximum deviation from this value is $R\equiv 3/7r_{0}$.

Non-bonded monomers separated by a distance less than $r_{c}\equiv 2.5\sigma$ interact according to the Lennard–Jones (LJ) potential, which is provided in Equation (2) [30]. The Lennard–Jones potential has a minimum when monomers are separated by a distance $r_{0}$. To achieve this, we use a parameter $\sigma \equiv 2^{-1/6}r_{0}$. In order to avoid a discontinuity in energy at $r_{c}$, we shift the potential by $v_{c}=4\left[{\left(\sigma /r_{c}\right)}^{12}-{\left(\sigma /r_{c}\right)}^{6}\right]$. The bonded and non-bonded interactions are given by

(1) $$\begin{array}{cc} v_{\mathrm{FENE}}\left(r\right) & =-\log\{1-{\left[\left(r-r_{0}\right)/R\right]}^{2}\} \end{array}$$

(2) $$\begin{array}{cc} v_{\mathrm{LJ}}\left(r\right) & =4\left[{\left(\sigma /r\right)}^{12}-{\left(\sigma /r\right)}^{6}\right]-v_{c} \end{array}$$

The widely used cosine-based semiflexible homopolymer model is parameterized as $v\left(\theta \right)=-\cos\left(\theta -\theta_{0}\right)$ [31,32]. We use a reference bending angle of $\theta_{0}=0$. With this choice, there is an energy penalty for any deviation from the straight chain configuration. In this paper, we introduce a generalization of this formulation that incorporates an angular width parameter, *w* which controls the range of angular offsets over which the bending potential is applied. This modification allows for tunable flexibility in the polymer backbone, affecting both structural stability and conformational variability. The modified bending potential is given by

(3) $$v_{\theta }\left(\theta \right)=\left\{\begin{array}{cc} -w\cos(1/w\left(\theta -\theta_{0}\right)) & \mathrm{if}\;|\theta -\theta_{0}|<\pi w,\\ w & \mathrm{otherwise}. \end{array}\right.$$

**Figure 1:**
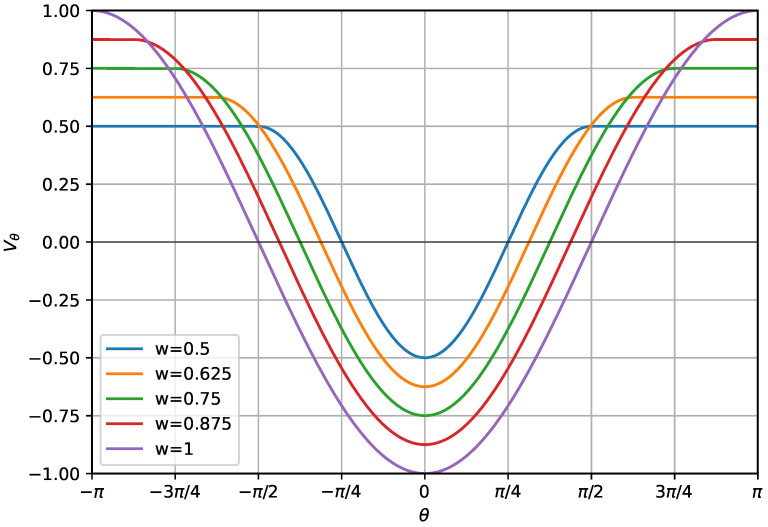
Plot of $v_{\theta }$ as a function of θ for κ=1. Curves are shown for values of *w* ranging between 0.5 and 1. Note that the maximum gradient of $v_{\theta }$ is consistent across for all values of *w* due to the scaling of the potential.

The inclusion of the angular width factor, *w*, determines the width of the potential well, as shown in Figure 1. When w=1, this model reduces to the standard semiflexible model. Choosing a smaller value of *w* narrows the width of the cosine, causing energetic penalties to take effect for smaller deviations from $\theta_{0}$. When comparing systems with different values of *w*, we find it useful to scale $v_{\theta }$ by *w* to maintain an equivalent maximum gradient of the potential. In exploratory simulations without this scaling, structural comparisons across different *w* values were less direct. We empirically observed that including the scaling factor facilitated a more direct practical comparison of the structures generated under varying angular widths. Introducing the angular width parameter allows for the simulation of a wider range of systems exhibiting qualitatively distinct structural behavior.

The total potential energy of a particular polymer configuration X can be calculated using the Hamiltonian provided in Equation (4). The Hamiltonian consists of all three potentials applied across the entire polymer, with each term being weighted by an appropriate energy scale. We use $S_{\mathrm{FENE}}=\frac{1}{2}KR^{2}$ with $K=\left(98/5\right)r_{0}^{2}$ and $S_{\mathrm{LJ}}=1$ for the FENE and LJ potentials. In this study, the values of $S_{\theta }$ vary linearly between a minimum value of $S_{\theta ,\min}=8$ and a maximum value of $S_{\theta ,\max}=19$. This provides a sufficient range for observing distinct structural transitions. These values were selected to span a broad range of structural behaviors, based on exploratory simulations.

(4) $$\begin{array}{cc} H(X) & =S_{\mathrm{LJ}}\sum_{i>j}v_{\mathrm{LJ}}\left(r_{ij}\right)+\\ \; & S_{\mathrm{FENE}}\sum_{i}v_{\mathrm{FENE}}\left(r_{i\;i+1}\right)+S_{\theta }\sum_{k}v_{\theta }\left(\theta_{k}\right) \end{array}$$

### 2.2. Parallel Tempering

All simulations in this paper consist of an array of simulation threads, with each thread generating a canonical ensemble at temperature *T* and obeying Hamiltonian *H*. Threads independently proceed with Monte Carlo updates. Each update changes the energy of the structure by ΔE. Any update that separates two bonded monomers by more than $r_{0}+R$ or brings them closer than $r_{0}-R$ is immediately rejected. Otherwise, updates are accepted with probability $P_{\mathrm{acc}}=\min\left(1,e^{-\beta \Delta E}\right)$, where $\beta =1/k_{B}T$. We use units in which $k_{B}\equiv 1$.

Most Monte Carlo updates consist of single monomer displacements in which a random monomer is chosen and then displaced to a random location within a box surrounding its original location. To enhance sampling efficiency, additional update moves include the collective displacement of a randomly chosen group of monomers and the rotation of a polymer segment around an axis perpendicular to adjacent bonds, modifying a single bend angle.

This study implements a two-dimensional replica-exchange Monte Carlo technique, also known as two-dimensional parallel tempering. Each thread attempts temperature exchanges with its neighboring threads at higher and lower temperatures, as well as Hamiltonian exchanges with neighboring threads with larger or smaller values of $S_{\theta }$. In this way, replicas perform a two-dimensional random walk in the $S_{\theta }$-*T* space, allowing for the efficient sampling of threads across both temperature and bending energy scales.

Exchanges are attempted every 500 Monte Carlo steps. In an exchange, two neighboring threads attempt to swap configurations, where thread *i* proposes sending structure $X_{i}$ to thread *j* while receiving structure $X_{j}$ in return. The exchange is accepted with probability

(5) $$P_{\mathrm{exch}}=\min\left(1,\frac{e^{\beta_{i}H_{i}\left(X_{i}\right)}e^{\beta_{j}H_{j}\left(X_{j}\right)}}{e^{\beta_{i}H_{i}\left(X_{j}\right)}e^{\beta_{j}H_{j}\left(X_{i}\right)}}\right).$$

In the case of a temperature exchange, $H_{i}=H_{j}\equiv H$ and Equation (5) becomes

(6) $$P_{\mathrm{exch},T}=\min\left(1,e^{\left(\beta_{i}-\beta_{j}\right)\left(H\left(X_{i}\right)-H\left(X_{j}\right)\right)}\right).$$

For the Hamiltonian exchange, $\beta_{i}=\beta_{j}\equiv \beta$ and Equation (5) becomes

(7) $$P_{\mathrm{exch},H}=\min\left(1,e^{\beta \left(H_{i}\left(X_{i}\right)+H_{j}\left(X_{j}\right)-H_{i}\left(X_{j}\right)-H_{j}\left(X_{i}\right)\right)}\right).$$

In this study, variations in the Hamiltonian *H* correspond to changes in the bending energy scale factor, $S_{\theta }$. The temperature values *T* are logarithmically spaced from $T_{\min}=0.2$ to $T_{\max}=1.6$, facilitating efficient sampling across a broad thermal spectrum. For each set of $S_{\theta }$ and *T*, simulations are performed for values of *w* ranging from 0.5 to 1.0, where w=1.0 recovers the standard semiflexible polymer model.

## 3. Results

### 3.1. Structure Types

**Figure 2:**
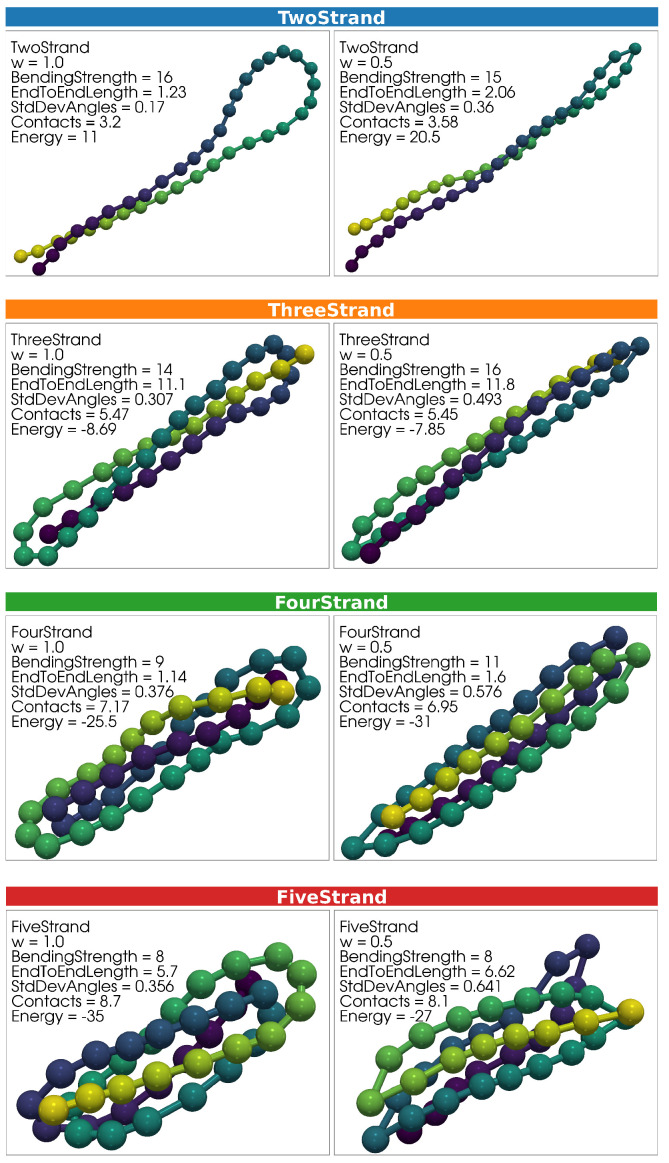
Representative structures for Two-Strand, Three-Strand, Four-Strand, and Five-Strand hairpin structures are shown. For each structure type, examples from both the w=1.0 and w=0.5 simulations are displayed. Within each panel, $S_{\theta }$, $L_{e2e}$, *C*, and *E* are indicated. While these examples illustrate general structural characteristics, the structural variability within each type is broader than what is captured in this graphic. Rectangles labeling structural representations are colored according to structure typs, consistent with the point-color scheme in Figures 5–7. Monomers within each polymer structure are colored according to a gradient solely to visually distinguish overlapping strands. Colors do not indicate any additional quantitative property.

**Figure 3:**
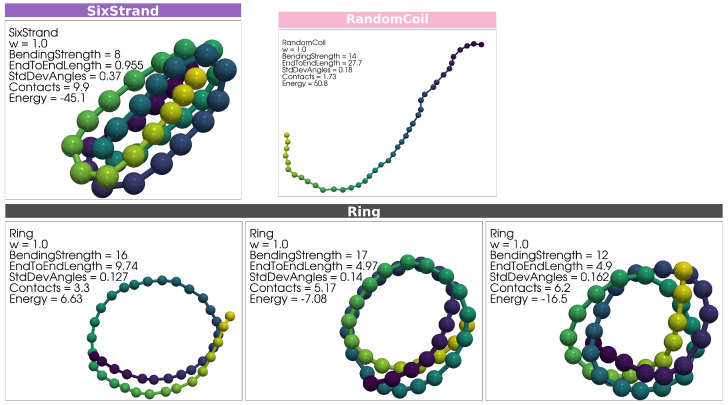
A representative Six-Strand hairpin structure from the w=1.0 simulation is shown. No Six-Strand structures were produced in the w=0.5 simulations. A representative Random Coil structure from the w=1.0 simulation is shown. Without systematic variation with *w*, we did not feel it necessary to include a w=0.5 example. Ring structures from each of the distinct Ring clusters identified in Figure 5b are displayed in the second row. Within each panel, the bending strength, End-to-End Length, Average Number of Contacts, and energy are indicated. While these examples illustrate the general structural characteristics, the structural variability within each type extends beyond what is captured in this figure. Rectangles labeling structural representations are colored according to structure types, consistent with the point-color scheme in Figures 5–7. Monomers within each polymer structure are colored according to a gradient solely to visually distinguish overlapping strands. Colors do not indicate any additional quantitative property.

A variety of distinct structural conformations are produced in each simulation. Representative configurations of key structural types are presented in Figure 2 and Figure 3. Example configurations for Two-Strand, Three-Strand, Four-Strand, and Five-Strand hairpin structures are shown in Figure 2, with one representative structure from the w=1.0 simulation and one from the w=0.5 simulation placed side by side for comparison. Figure 3 includes Six-Strand structures and Ring structures from the w=1.0 simulation. Ring structures were not observed for w=0.5, as discussed previously.

Structural variability extends beyond what is depicted in these figures. Example structures were selected to be as close as possible to the center of their respective structural clusters, based on $\sigma_{\theta }$, *C*, $L_{e2e}$, and *E*.

The top row of Figure 2 presents two representative Two-Strand structures. The first example is from the w=1.0 simulation, and the second is from the w=0.5 simulation. The w=1.0 example structure features a broad, arcing joint that minimizes individual bending angle deviations from 0 degrees. The vertical extension of the Two-Strand structure cluster in Figure 5b reflects variability in joint types, ranging from the broad arcing joint shown here to a narrower joint more closely resembling the w=0.5 example structure. This variation results from a trade-off between maximizing the number of contacts and minimizing the total bending energy penalty. In contrast, all Two-Strand structures in the w=0.5 simulation exhibit narrow joints, where a single bending angle bears most of the bending penalty, allowing other angles to remain near 0 degrees.

Hairpin structures with more strands exhibit similar features to the Two-Strand case. As in the Two-Strand structures, joints in the w=1.0 simulations range from broad, arcing forms to narrow configurations, while the w=0.5 simulations exclusively produce structures with narrow joints. This variability in joint type is reflected in the vertical extension of clusters within Figure 5b, highlighting a continuum of bending energy trade-offs similar to those observed in the Two-Strand case.

One representative Six-Strand structure is shown in the top row of Figure 3. This structure was generated within the w=1.0 simulation, and no such structures were observed in the w=0.5 simulation. Multiple clusters of Ring structures were produced in the w=1.0 simulation. A single representative structure is selected from the center of each cluster in Figure 5b. As the number of wraps in a Ring structure increases, so does the number of contacts, with successive clusters in Figure 5b representing increasingly wrapped conformations along the horizontal axis.

### 3.2. Microcanonical Analysis

To identify structural transitions from the simulation results, we locate inflection points in the microcanonical entropy and its derivatives. The microcanonical entropy, defined as $S\left(E\right)=k_{B}\log\left(g\left(E\right)\right)$, is calculated by reweighting the histograms produced by individual simulation threads. Each simulation thread *i*, operating at temperature *T*, generates a histogram $h_{i}\left(E\right)$, which estimates the density of states as ${\bar{g}}_{i}\left(E\right)=h_{i}\left(E\right)e^{\beta E}$. The statistical accuracy of this estimate at a given energy value *E* depends on the number of histogram counts in that energy bin, with more counts leading to improved reliability.

If histograms from threads at different temperatures overlap sufficiently in terms of energy space, they can be weighted to produce a density of states, g(E), across the full range of *E* [33]. To achieve this, we initialize the partition function with Z=1 at all temperatures. An estimate of $\hat{g}\left(E\right)$ is then computed using Equation (8), where $M_{i}$ represents the total number of energy samples (histogram counts) from thread *i*. This estimate normalizes the histogram data across threads, enabling an approximate and consistent density of the states. The partition function estimate is then iteratively refined using Equation (9) until convergence.

(8) $$\hat{g}\left(E\right)=\frac{\sum_{i}h_{i}\left(E\right)}{\sum_{i}M_{i}Z_{i}^{-1}e^{-\beta_{i}E}}.$$

(9) $$Z_{i}=\sum_{E}\hat{g}\left(E\right)e^{-\beta_{i}E}.$$

From the density of states, the microcanonical entropy can be calculated. Bezier smoothing is applied to reduce statistical noise in *S* and improve numerical stability when computing its derivatives while preserving underlying trends [34]. The derivatives of S(E), denoted as β(E)≡dS/dE, $\gamma \left(E\right)\equiv d^{2}S/dE^{2}$, and $\delta \left(E\right)\equiv d^{3}S/dE^{3}$, are used to analyze structural transitions.

An $n^{\mathrm{th}}$-order structural transition in a finite system appears as a region of reduced variation in the ${\left(n-1\right)}^{\mathrm{th}}$ derivative of the microcanonical entropy, leading to a zero crossing in the ${\left(n+1\right)}^{\mathrm{th}}$ derivative of the entropy. These zero crossings correspond to an inflection point that is two orders lower, which serves as an indicator of a structural transition [35].

**Figure 4:**
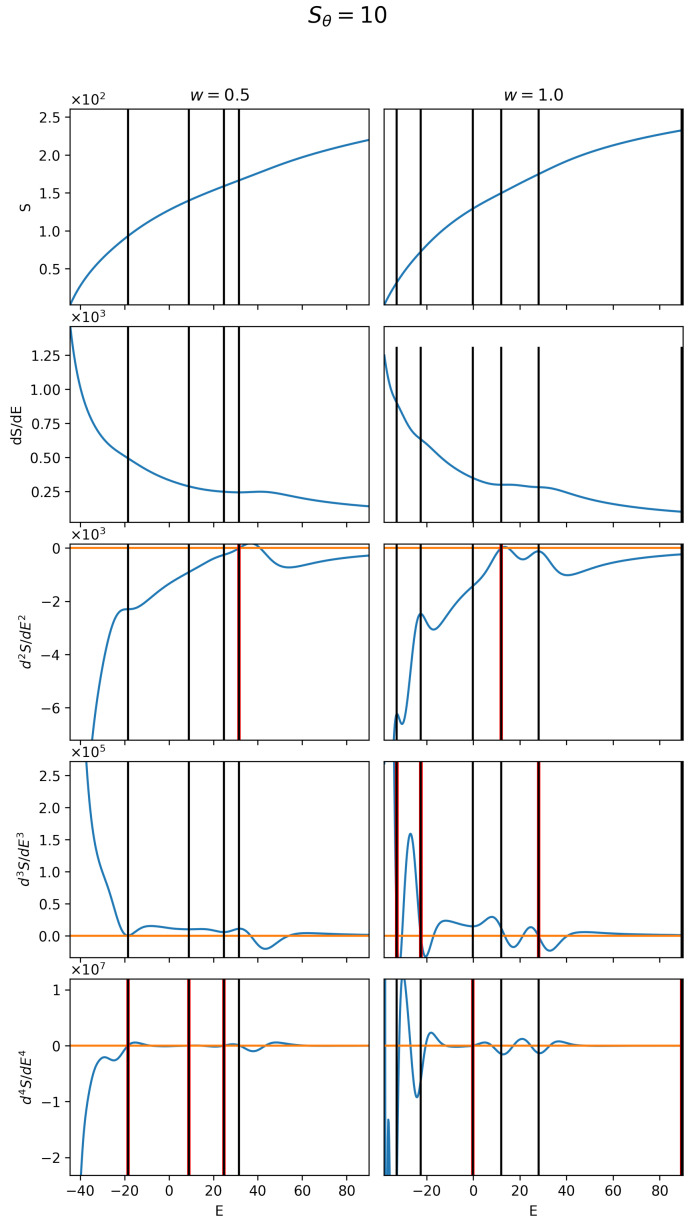
Example plots of the first four derivatives of *S* for bending strength of $S_{\theta }=10$. The left column corresponds to w=0.5, and the right w=1.0. Zero lines are shown in orange, and vertical red lines indicate zero crossings in the derivatives that correspond to structural transitions. Vertical black lines mark the transition energies, aligning these crossings across all derivative orders.

Figure 4 provides example plots of the entropy and its first four derivatives for a bending strength of $S_{\theta }=10$. Most of the structural transitions identified using this method align well with the qualitative changes in structure type, as described in Section 3.3.

In the w=0.5 case, four distinct structural transitions of orders 1–3 are identified. A first-order structural transition occurs at approximately E=30, indicated by an inflection point in the entropy curve, a peak in the first derivative, and a zero crossing in the second derivative. Three third-order structural transitions appear as regions of reduced variation in the second derivative, dips in the third derivative, and zero crossings in the fourth derivative, occurring at approximately E=−19, 9, and 23. While further derivatives could be used to identify fourth-order transitions, distinguishing qualitatively distinct structures at such higher orders would require additional structural analysis.

For the w=1.0, five structural transitions of orders 1–3 are identified. A first-order structural transition occurs at E=12, marked by an inflection point in the entropy curve, a slight peak in the first derivative, and a zero crossing in the second derivative. Three second-order structural transitions, indicated by regions of reduced variation in the first derivative and zero crossings in the third derivative, occur at E=−33,−21,and29. A single third-order transition is observed at E=0, characterized by a region of reduced variation in the second derivative and a zero crossing in the fourth derivative.

### 3.3. Structural Classification and Diagram Comparison

**Figure 5:**
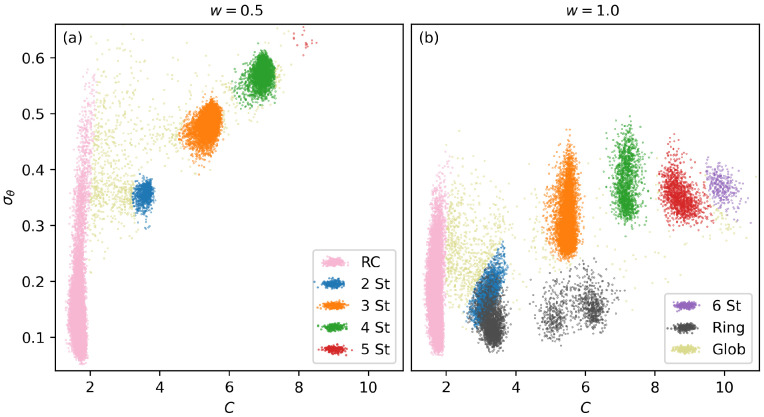
Structural classification based on clustering within the $\sigma_{\theta }$-*C* structural parameter space is illustrated, alongside the structure representation across temperature and energy, for polymers of length 40 and w=1.0. (**a**,**b**) display scatter plots of structural clusters for w=0.5 and w=1.0, respectively. The color key for structure types used throughout this paper is shown in these two panels.

Structures are classified by analyzing the clustering of example structures across an array of carefully chosen structural parameters. The primary parameters used to distinguish structures are the standard deviation of bending angles ($\sigma_{\theta }$) and the per-monomer number of contacts (*C*). Scatter plots illustrating the structures produced in selected simulation runs are presented in Figure 5. Each panel represents a sampling of structures from every thread of a single two-dimensional parallel tempering simulation. Structure clusters are identifiable through visual inspection. Three-dimensional models of representative structures within each cluster are examined, and all structures are labeled according to their respective clusters. While some clusters overlap in the two-dimensional representations shown here, they can be distinguished by considering their positions in additional structural parameters, such as energy (*E*) and end-to-end length ($L_{e2e}$). This multi-parameter classification helps ensure that structural distinctions are robust. Three-dimensional representations of example structures from each cluster are presented in Section 3.1.

**Figure 6:**
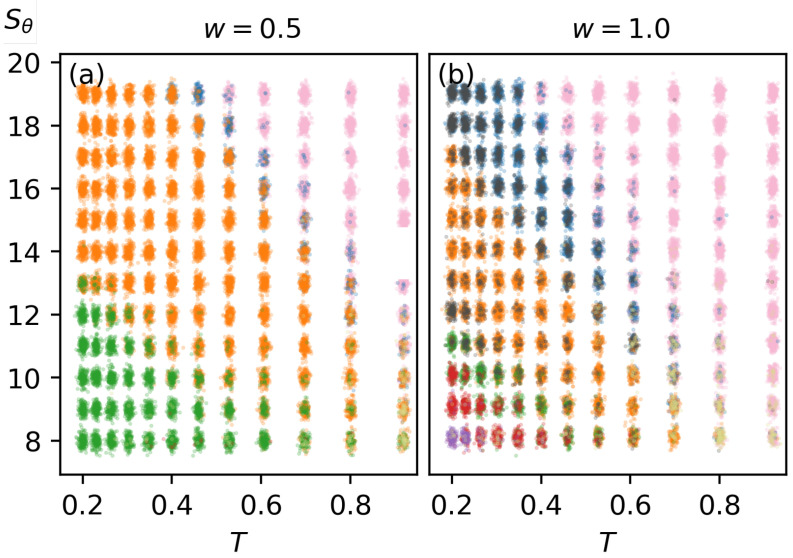
Structural transition diagram showing structure distribution across bending strength ($S_{\theta }$) and temperature (*T*). Colors representing each structure type are the same as in Figure 5. (**a**,**b**) correspond to w=0.5 and w=1.0, respectively.

**Figure 7:**
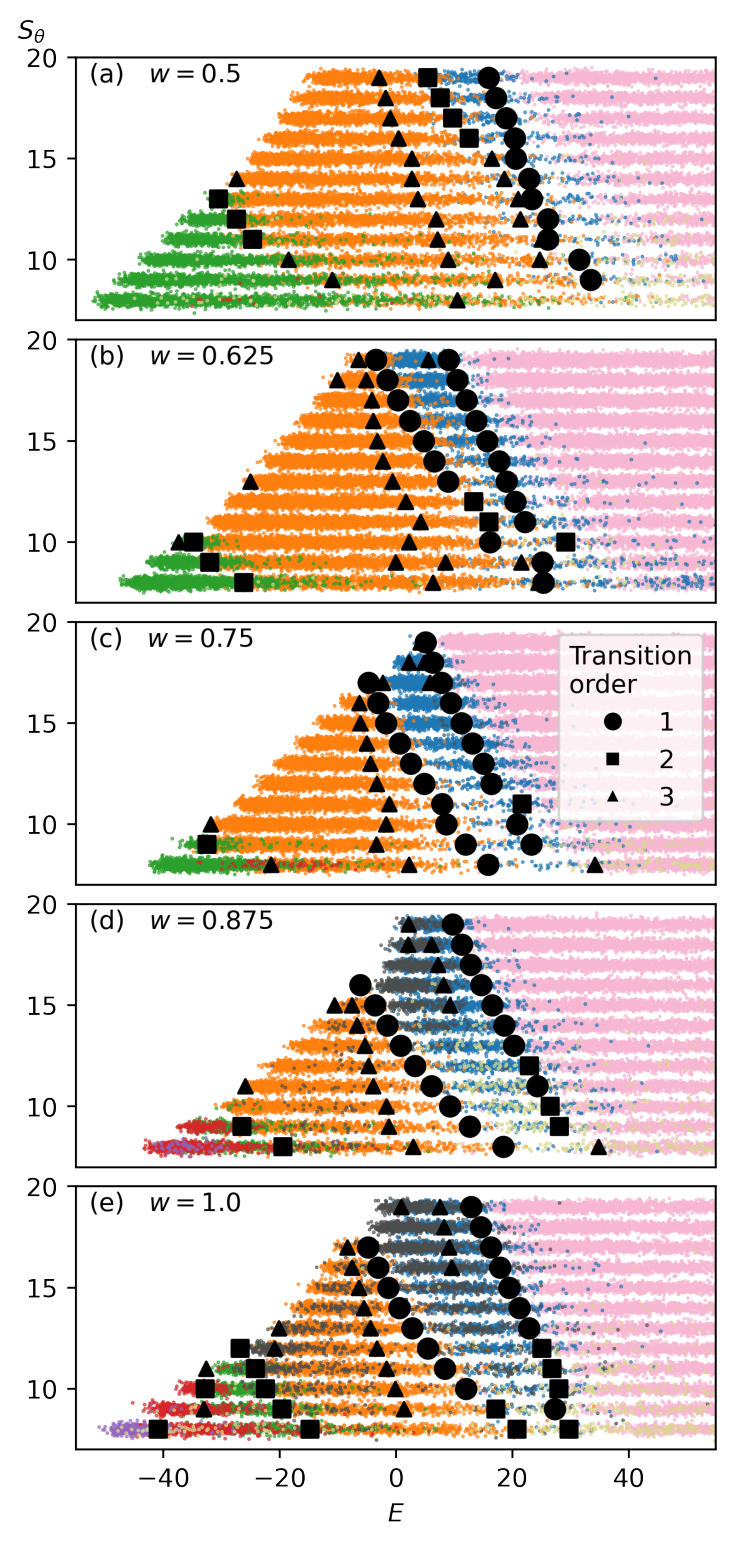
Microcanonical structural transition diagram, plotting structures as a function of bending strength ($S_{\theta }$) and energy (*E*), Transition points are marked by circles for first-order transitions, squares for second-order transitions, and triangles for third-order transitions. The colors representing each structure type are the same as in Figure 5.

Once the structures are classified according to their clustering within structural parameter space, they are plotted as a function of $S_{\theta }$ and *T*. Figure 6 illustrates this representation. To mitigate overlapping points, we apply Gaussian-distributed jitter, which spreads the points and provides a clearer depiction of each structure type’s distribution. This approach effectively creates a canonical structural transition diagram. Similarly, a microcanonical representation is generated, as shown in Figure 7, by plotting structures according to $S_{\theta }$ and *E*. Microcanonical structural transitions are also included in this representation.

#### 3.3.1. w=1.0 Simulation

In the w=1.0 simulation, eight distinct structure types are represented. Random Coil (RC) structures are flexible, single-strand linear polymers that do not exhibit a persistent structural organization. Hairpin structures, characterized by multiple relatively straight strands connected by bends or joints, are labeled Two-Strand through Six-Strand (2 St – 6 St). The bends connecting the strands can vary in sharpness, influencing overall polymer shape. Ring structures, where the polymer wraps around itself with overlapping ends, are divided into several distinct clusters based on the degree of overlap. For the purposes of this paper, we do not distinguish between rings with differing degrees of overlap. Finally, Globules (Glob) exhibit a dense, disordered arrangement with significant contacts between non-neighboring monomers but lack a persistent secondary structure or long-range order.

In both the canonical and microcanonical representations, significant structural coexistence can be observed. This occurs among various Hairpin structures, where the probability of one structure type gradually decreases as another becomes more dominant. Ring structures are present across a broad range of structural space but typically appear as a minority within most structural regimes.

First-, second-, and third-order structural transitions from the microcanonical analysis are identified for the w=1.0 simulations. Strong first-order transitions, characterized by sharp entropy changes, are consistently observed between the Random Coil state and the Two-Strand state, as well as between the Two-Strand and Three-Strand states. For some $S_{\theta }$ values, particularly at weaker bending strengths, these transitions are classified as second-order instead of first-order. A consistently second-order transition is observed between the Three-Strand and Four-Strand states, as well as between the Five-Strand and Six-Strand states. A mixture of second- and third-order transitions occurs between the Four-Strand and Five-Strand states. At a $S_{\theta }=8$, Four-Strand structures are not sufficiently dominant, and no clear transition to Five-Strand structures is observed.

Additionally, third-order structural transitions are identified in several regions. One occurs between the region dominated by Two-Strand structures and the low-energy, high-bending-strength region where Ring structures dominate. Another consistent third-order transition is found on the high-energy side of the Three-Strand structural state, though no clear structural change has been identified at this transition, suggesting the need for further analysis. Finally, in the low-energy, high-bending-strength region of the Three-Strand structural state, a third-order transition to Ring structures is observed.

#### 3.3.2. w=0.5 Simulation

The structure types observed in the w=0.5 results are largely similar to those in the w=1.0 simulation, with the notable exception that Ring structures are entirely absent, as the narrowed bending potential suppresses their formation. In Figure 5, more distinct clustering of structure types is observed, with tighter clusters and fewer ambiguous structures bridging distinct structural states. Additionally, there is significantly more structural separation within both the canonical and microcanonical representations. Structural coexistence between various Hairpin structures is reduced and Ring structures do not appear in this regime.

Two-Strand structures dominate a significantly smaller region of the *T* vs. $S_{\theta }$ structural space compared to the w=1.0 simulation. Extending the w=0.5 simulation to higher bending strengths may further expand the structural space where Two-Strand structures are dominant, warranting further investigation.

Due to the additional structural stability provided by the narrowed bending restraint in the w=0.5 case, Four-Strand structures occupy a significantly larger region of structural space compared to the w=1.0 case. This expansion entirely displaces the Five-Strand and Six-Strand structures from dominating any region of structural space for w=0.5. In contrast, for w=1.0, there is a low-temperature, low-bending-strength region where Five-Strand structures dominate. In the w=0.5 simulation, Five-Strand structures were extremely rare and did not form a dominant structural region. However, with an expanded simulation space that includes lower bending strengths and temperatures, it is possible that a distinct Five-Strand structural region could develop.

Similar to the w=1.0 case, we observe a consistent first-order transition between the Random Coil structural region and the Two-Strand structural region, extending even to areas where Two-Strand structures are not dominant. The transition between the dominant Two-Strand and Three-Strand structural regions is second-order. As bending strength decreases, this transition shifts to third-order. We again observe a third-order transition within the middle of the Three-Strand structural region, though no apparent structural change is detected at this transition, suggesting the need for further analysis. Between Three-Strand and Four-Strand structures, we see either second- or third-order transitions, depending on bending strength. While we can observe a sparse representation of Five-Strand structures, we have not explored sufficiently low bending strengths to identify a region where Five-Strand structures dominate.

#### 3.3.3. Intermediate Values of *w*

Microcanonical structural space plots for intermediate values of *w* are shown in Figure 7. As *w* increases, the Three-Strand structural region progressively shrinks, leading to a broader distribution of structures across both high and low bending-strength values. At high bending strengths, the Two-Strand structural region expands, while at lower bending strengths, Five-Strand and Six-Strand structures become more prominent.

With increasing *w*, there is also a progressive increase in structural coexistence. This is evident both between neighboring structural regions and in the increased presence of Ring structures across multiple structural regions, particularly in the w=0.875 and w=1.0 cases. This increase in structural coexistence corresponds to the decreased structural stability for all low-temperature structures under the conditions of a wider bending restraint. The broader distribution of structures underscores the role of *w* in modulating structural diversity and stability across temperature and energy spaces.

## 4. Discussion

This study demonstrates the diverse array of semiflexible polymer behaviors that emerge from introducing an angular width parameter (*w*) into the bending potential. We find that this parameter strongly influences the range of structural states observed, the degree of structural coexistence, and the structural variability of semiflexible polymers. Through a combination of canonical and microcanonical analyses, we identified a range of structural states, including Two-Strand, Ring, and Globule structures, and their dependence on bending strength and temperature. Narrowing the bending potential (w=0.5) increases structural stability while reducing structural coexistence and variability. The increased structural stability leads to the Four-Strand structural state dominating over other structure types, such as the Five-Strand, Six-Strand, and Ring structures observed in the w=1.0 simulation. Additionally, the expansion of the Three-Strand structural state significantly reduces the structural space representation of Two-Strand structures.

The first four derivatives of the microcanonical entropy reveal clear markers of first-, second-, and third-order structural transitions, offering deeper insight into the thermodynamic properties of these systems. These findings have important implications for understanding the self-assembly and structural behavior of polymers under physical constraints, with potential applications in nanomedicine, materials science, and soft matter physics.

Future work could explore several promising avenues to extend and deepen the current analysis. First, simulations could cover a broader range of bending strengths, to more clearly capture multistrand bundle formation, especially in cases of narrower bending potentials. Additionally, extending the simulations over a wider temperature range would enable the clearer identification of structural transitions and their thermodynamic characteristics. Exploring smaller values for the angular width parameter could provide valuable insights as the bending potential approaches the fully flexible (freely jointed) polymer limit. Furthermore, performing canonical analyses to investigate structural transitions based on temperature-dependent properties, such as the specific heat, would complement the microcanonical findings presented here. Finally, comparing our coarse-grained results to more applied polymer systems could strengthen the relevance of this new potential for practical polymer modeling.

## Data Availability

The data that support the findings of this study are available from the corresponding author upon reasonable request.

## References

[1] Kmiecik S., Gront D., Kolinski M., Wieteska L., Dawid A.E., Kolinski A. (2016). Coarse-grained protein models and their applications. Chem. Rev..

[2] Yuan C., Chen H., Lou X.W., Archer L.A. (2008). DNA bending stiffness on small length scales. Phys. Rev. Lett..

[3] Sluysmans D., Willet N., Thevenot J., Lecommandoux S., Duwez A.-S. (2020). Single-molecule mechanical unfolding experiments reveal a critical length for the formation of α-helices in peptides. Nanoscale Horiz..

[4] Wu J., Cheng C., Liu G., Zhang P., Chen T. (2018). The folding pathways and thermodynamics of semiflexible polymers. J. Chem. Phys..

[5] Gao P., Nicolas J., Ha-Duong T. (2021). Supramolecular organization of polymer prodrug nanoparticles revealed by coarse-grained simulations. J. Am. Chem. Soc..

[6] Ferreira L.G., Santos R.N.D., Oliva G., Andricopulo A.D. (2015). Molecular docking and structure-based drug design strategies. Molecules.

[7] Katzgraber H.G., Trebst S., Huse D.A., Troyer M. (2006). Feedback-optimized parallel tempering Monte Carlo. J. Stat. Mech..

[8] Singh N., Li W. (2019). Recent advances in coarse-grained models for biomolecules and their applications. Int. J. Mol. Sci..

[9] Bustamante C., Marko J.F., Siggia E.D., Smith S. (1994). Entropic elasticity of *λ*-phage DNA. Science.

[10] Kremer K., Grest G.S. (1990). Dynamics of entangled linear polymer melts: A molecular-dynamics simulation. J. Chem. Phys..

[11] Aierken D., Bachmann M. (2023). Impact of bending stiffness on ground-state conformations for semiflexible polymers. J. Chem. Phys..

[12] Lifshitz I.M., Grosberg A.Y., Khokhlov A.R. (1978). Some problems of the statistical physics of polymer chains with volume interaction. Rev. Mod. Phys..

[13] Grosberg A.Y., Khokhlov A.R., Stanley H.E., Mallinckrodt A.J., McKay S. (1995). Statistical physics of macromolecules. Comput. Phys..

[14] Williams M.J., Bachmann M. (2016). Significance of bending restraints for the stability of helical polymer conformations. Phys. Rev. E.

[15] Williams M.J. (2023). Microcanonical Analysis of Helical Homopolymers: Exploring the Density of States and Structural Characteristics. Polymers.

[16] Zierenberg J., Marenz M., Janke W. (2016). Dilute semiflexible polymers with attraction: Collapse, folding and aggregation. Polymers.

[17] Junghans C., Bachmann M., Janke W. (2009). Statistical mechanics of aggregation and crystallization for semiflexible polymers. Europhys. Lett..

[18] Milchev A., Binder K. (2020). Semiflexible polymers interacting with planar surfaces: Weak versus strong adsorption. Polymers.

[19] Sintes T., Sumithra K., Straube E. (2001). Adsorption of semiflexible polymers on flat, homogeneous surfaces. Macromolecules.

[20] Martínez-Fernández D., Herranz M., Foteinopoulou K., Karayiannis N.C., Laso M. (2023). Local and global order in dense packings of semi-flexible polymers of hard spheres. Polymers.

[21] Milchev A., Egorov S.A., Binder K., Nikoubashman A. (2018). Nematic order in solutions of semiflexible polymers: Hairpins, elastic constants, and the nematic-smectic transition. J. Chem. Phys..

[22] Shakirov T., Paul W. (2018). Crystallization in melts of short, semiflexible hard polymer chains: An interplay of entropies and dimensions. Phys. Rev. E.

[23] Milchev A., Egorov S.A., Vega D.A., Binder K., Nikoubashman A. (2018). Densely packed semiflexible macromolecules in a rigid spherical capsule. Macromolecules.

[24] Kimura K., Higuchi S. (2016). Extension of the constant exchange probability method to multi-dimensional replica exchange Monte Carlo applied to the tri-critical spin-1 Blume–Capel model. J. Stat. Mech..

[25] Mitsutake A., Okamoto Y. (2009). From multidimensional replica-exchange method to multidimensional multicanonical algorithm and simulated tempering. Phys. Rev. E.

[26] Fukunishi H., Watanabe O., Takada S. (2002). On the Hamiltonian replica exchange method for efficient sampling of biomolecular systems: Application to protein structure prediction. J. Chem. Phys..

[27] Schnabel S., Seaton D.T., Landau D.P., Bachmann M. (2011). Microcanonical entropy inflection points: Key to systematic understanding of transitions in finite systems. Phys. Rev. E.

[28] Qi K., Bachmann M. (2018). Classification of phase transitions by microcanonical inflection-point analysis. Phys. Rev. Lett..

[29] Bird R.B., Armstrong R.C., Hassager O., Bird R.B. (1987). Dynamics of Polymeric Liquids. 2: Kinetic Theory.

[30] Qi K., Liewehr B., Koci T., Pattanasiri B., Williams M.J., Bachmann M. (2019). Influence of bonded interactions on structural phases of flexible polymers. J. Chem. Phys..

[31] Marenz M., Janke W. (2016). Knots as a topological order parameter for semiflexible polymers. Phys. Rev. Lett..

[32] Aierken D., Bachmann M. (2023). Secondary-structure phase formation for semiflexible polymers by bifurcation in hyperphase space. Phys. Chem. Chem. Phys..

[33] Conrad P.B., de Pablo J.J. (1998). Comparison of histogram reweighting techniques for a flexible water model. Fluid Ph. Equilib..

[34] Bachmann M. (2014). Thermodynamics and Statistical Mechanics of Macromolecular Systems.

[35] Sitarachu K., Bachmann M. (2022). Evidence for additional third-order transitions in the two-dimensional Ising model. Phys. Rev. E.

